# Supplementary material for: Probing topological protection using a designer surface plasmon structure
Source: Nat Commun. 2016 May 20;7:11619. doi: 10.1038/ncomms11619 (PMC4876474; doi:10.1038/ncomms11619)
Supplement: Supplementary Information — Supplementary Figures 1-3 and Supplementary Notes 1-3. [file ncomms11619-s1.pdf]

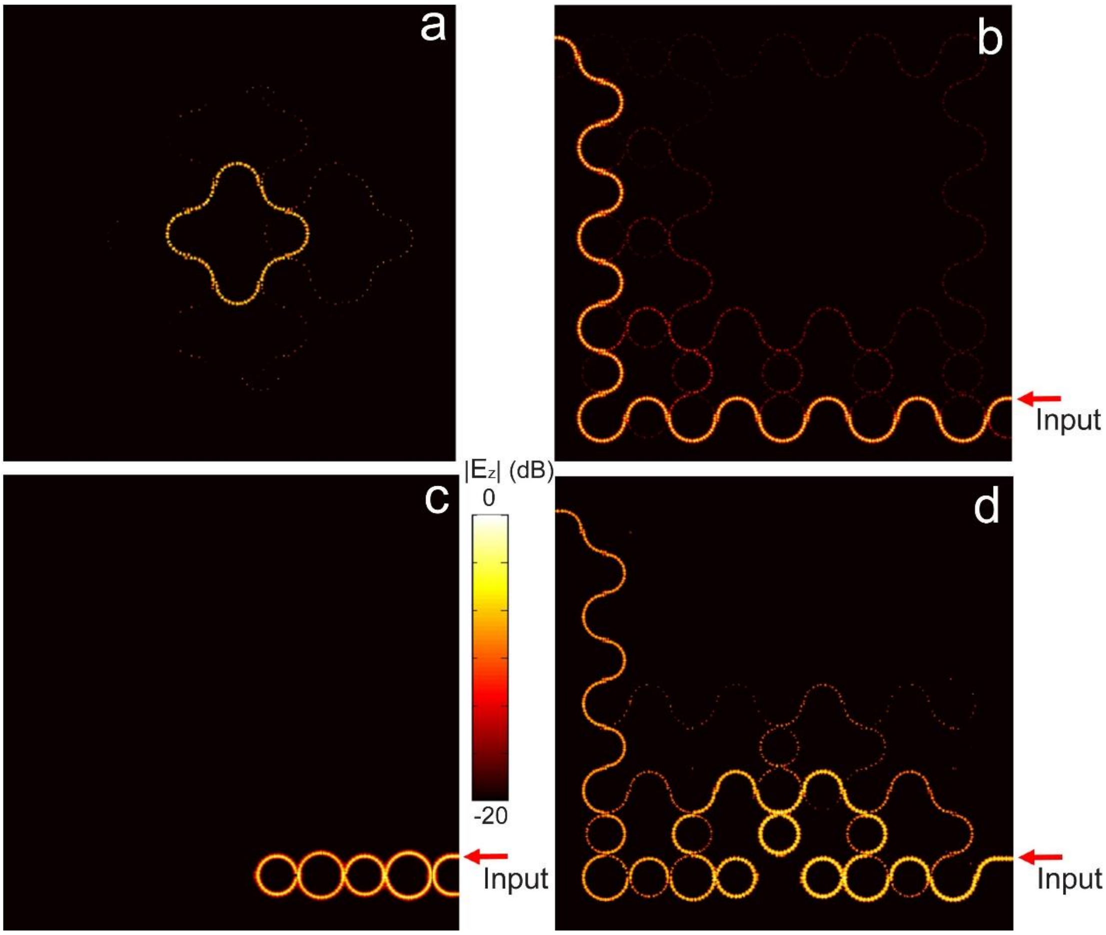

Supplementary Figure 1 | Simulated field patterns according to experimental results in Fig. 4. a, An insulating bulk state, corresponding to Fig. 4b. **b,** A topological edge state, corresponding to Fig. 4c. **c,** States on a 1D lattice, corresponding to Fig. 4e. **d,** Circumvention of the topological edge state around a defect ring with 3.5mm height, corresponding to Fig. 4g.

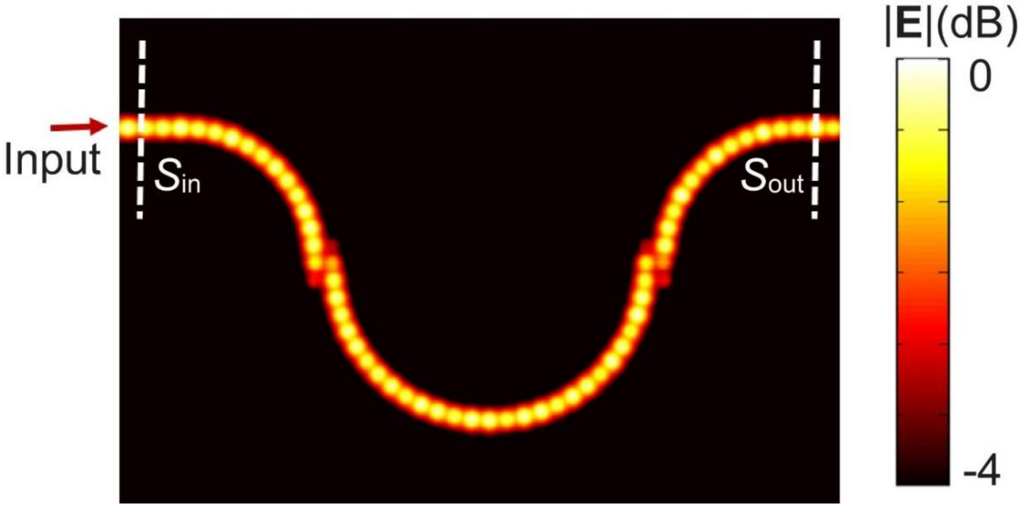

Supplementary Figure 2 | Simulation of coupling through a unit cell in the topological designer surface plasmon structure. The frequency is at 11.3 GHz.

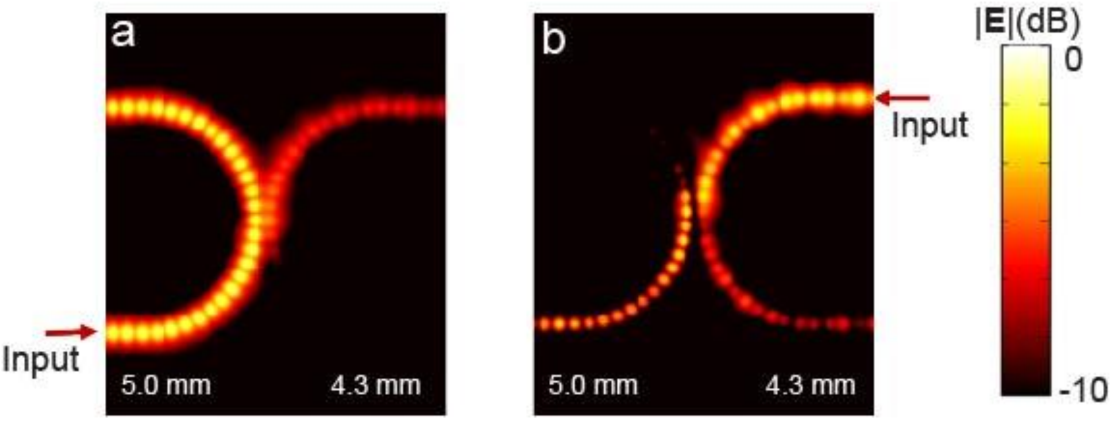

Supplementary Figure 3 | Simulation of coupling between a 5-mm-tall ring to a 4.3-mm-tall ring. The frequency is at 11.3 GHz. **a**, Input from the 5-mm-tall ring. **b**, Input from the 4.3 mm-tall ring.

Supplementary Note 1. Transfer Matrix Analysis and Topological Transition

The schematic of a unit cell for the square lattice network is shown in Fig. 2 in the main text. Here we give detailed calculations on band structure. Let $n \equiv (x_n, y_n)$ denote the site of unit cell. In each unit cell, input amplitudes can be described by a four-vector $(a_{1,n}, a_{2,n}, a_{3,n}, a_{4,n})$, and output amplitudes by another four-vector $(b_{1,n}, b_{2,n}, b_{3,n}, b_{4,n})$. The coupling between the sites at n and $n+x$ can be described as $\begin{bmatrix} a_{2,n} \\ a_{4,n+x} \end{bmatrix} = S_{nx} \begin{bmatrix} b_{1,n} \\ b_{3,n+x} \end{bmatrix}$ and the coupling between the sites at n and $n+y$ can be described as $\begin{bmatrix} a_{1,n} \\ a_{3,n+y} \end{bmatrix} = S_{ny} \begin{bmatrix} b_{4,n} \\ b_{2,n+y} \end{bmatrix}$, where $S_{nx} = S_{ny} = S_n = \begin{bmatrix} r & t' \\ t & r' \end{bmatrix}$ is a unitary matrix containing four complex numbers r,r',t and t'. Combining together, we can write down the following scattering matrix equation

$$\begin{bmatrix} a_{1,n} \\ a_{2,n} \\ a_{3,n+y} \\ a_{4,n+x} \end{bmatrix} = \begin{bmatrix} 0 & t' & 0 & r \\ r & 0 & t' & 0 \\ 0 & r' & 0 & t \\ t & 0 & r' & 0 \end{bmatrix} \begin{bmatrix} b_{1,n} \\ b_{2,n+y} \\ b_{3,n+x} \\ b_{4,n} \end{bmatrix} \quad (1)$$

This periodic network allows Bloch theorem to apply. We can thus get

$$\begin{bmatrix} a_{1,n} \\ a_{2,n} \\ a_{3,n+y} \\ a_{4,n+x} \end{bmatrix} = \begin{bmatrix} 1 & 0 & 0 & 0 \\ 0 & 1 & 0 & 0 \\ 0 & 0 & e^{iK_y} & 0 \\ 0 & 0 & 0 & e^{iK_x} \end{bmatrix} \begin{bmatrix} a_{1,n} \\ a_{2,n} \\ a_{3,n} \\ a_{4,n} \end{bmatrix}$$

$$\begin{bmatrix} b_{1,n} \\ b_{2,n+y} \\ b_{3,n+x} \\ b_{4,n} \end{bmatrix} = \begin{bmatrix} 1 & 0 & 0 & 0 \\ 0 & e^{iK_y} & 0 & 0 \\ 0 & 0 & e^{iK_x} & 0 \\ 0 & 0 & 0 & 1 \end{bmatrix} \begin{bmatrix} b_{1,n} \\ b_{2,n} \\ b_{3,n} \\ b_{4,n} \end{bmatrix} \quad (2)$$

After substituting Eq. (2) into Eq. (1), we can get:

$$\begin{bmatrix} a_{1,n} \\ a_{2,n} \\ a_{3,n} \\ a_{4,n} \end{bmatrix} = \begin{bmatrix} 1 & 0 & 0 & 0 \\ 0 & 1 & 0 & 0 \\ 0 & 0 & e^{-iK_y} & 0 \\ 0 & 0 & 0 & e^{-iK_x} \end{bmatrix} \begin{bmatrix} 0 & t' & 0 & r \\ r & 0 & t' & 0 \\ 0 & r' & 0 & t \\ t & 0 & r' & 0 \end{bmatrix} \begin{bmatrix} 1 & 0 & 0 & 0 \\ 0 & e^{iK_y} & 0 & 0 \\ 0 & 0 & e^{iK_x} & 0 \\ 0 & 0 & 0 & 1 \end{bmatrix} \begin{bmatrix} b_{1,n} \\ b_{2,n} \\ b_{3,n} \\ b_{4,n} \end{bmatrix} \quad (3)$$

By expressing $\begin{bmatrix} a_{1,n} \\ a_{2,n} \\ a_{3,n} \\ a_{4,n} \end{bmatrix} = e^{-i\phi} \begin{bmatrix} b_{1,n} \\ b_{2,n} \\ b_{3,n} \\ b_{4,n} \end{bmatrix}$ in Eq. (3), we rewrite the governing scattering matrix equation as:

$$S(K)|b_K\rangle = e^{-i\phi}|b_K\rangle \tag{4}$$

where $S(K) = \begin{bmatrix} 1 & 0 & 0 & 0 \\ 0 & 1 & 0 & 0 \\ 0 & 0 & e^{-iK_y} & 0 \\ 0 & 0 & 0 & e^{-iK_x} \end{bmatrix} \begin{bmatrix} 0 & t' & 0 & r \\ r & 0 & t' & 0 \\ 0 & r' & 0 & t \\ t & 0 & r' & 0 \end{bmatrix} \begin{bmatrix} 1 & 0 & 0 & 0 \\ 0 & e^{iK_y} & 0 & 0 \\ 0 & 0 & e^{iK_x} & 0 \\ 0 & 0 & 0 & 1 \end{bmatrix}$, $|b_K\rangle = \begin{bmatrix} b_{1,n} \\ b_{2,n} \\ b_{3,n} \\ b_{4,n} \end{bmatrix}$.

Following the same parameterization process in Ref. 20, the unitary scattering matrix $S_n = \begin{bmatrix} r & t' \\ t & r' \end{bmatrix}$ can be rewritten as $\begin{bmatrix} sin\theta e^{i\chi} & -cos\theta e^{i(\varphi-\xi)} \\ cos\theta e^{i\xi} & sin\theta e^{i(\varphi-\chi)} \end{bmatrix}$, where θ, representing the coupling strength between neighboring rings, is relevant with amplitudes of r and t, and χ, φ, ξ are relevant with phases of r and t. We choose $\chi = -0.24\pi, \varphi = \pi, \xi = 0$, extracted from simulated transmission of a unit cell at 11.3 GHz. The band structure of a semi-infinite strip with 50 lattices in *y* direction and periodic in *x* direction is plotted in Fig. 2c in the main text, where $\theta = 0.2\pi$ for weak coupling, $\theta = 0.25\pi$ for critical coupling, and $\theta = 0.4\pi$ for strong coupling, respectively.

Supplementary Note 2. Retrieval of coupling strength θ

Parameter θ describes coupling strength between neighboring lattice rings, which depends on both frequency ω, and ring-ring distance g. Topological nontrivial phase occurs above the critical $\theta = 0.25\pi$, while trivial phase occurs below this critical θ value. In real structures that support narrowband designer surface plasmons, we tune g to realize different topological phases. To match with band diagrams, we retrieved θ with $\theta = \text{asin}^{-1}[(I_{out}/I_{in})^{1/2}]$ from simulations on a unit cell, where I_{in} is the power delivered

into the unit cell through the input plane S_{in}, and I_{out} is the output power through the output plane S_{out}, as marked in Fig. S2. Topological edge state at 11.3 GHz has $\theta = 0.41\pi$ ($\pm 0.03\pi$ within the topological band gap), larger than 0.25π. Note that the coupling strength θ characterizes the inter-ring coupling, not the propagation loss. Therefore, the measured propagation loss of 1.44 dB per lattice constant is irrelevant here. Regarding the topologically trivial phase with *g* = 7.5 mm, the demonstrated bulk state at 11.3 GHz corresponds to $\theta = 0.10\pi$, smaller than 0.25π, and the in-gap excitation at 11.45 GHz corresponds to $\theta = 0.08\pi$, also smaller than 0.25π.

Supplementary Note 3. Coupling between a 5.0-mm ring and a 4.3-mm ring

In experiment, the 4.3-mm-tall ring is used as a defect and later used as the input waveguide. In Fig. S3, we simulate the coupling between a 5.0-mm-tall ring and a 4.3-mm-tall ring. We can see in Fig. S3a that, the wave coupled from the 5.0-mm-tall ring to the 4.3-mm-tall ring is minimal (~-7dB) and the wave maintains its propagation (~-1dB) on the 5.0-mm-tall ring without reflection. In Fig. S3b, the coupling from the 4.3-mm-tall ring to the 5.0-mm-tall ring is also weak (~-7dB). There is moderate radiation on the 4.3-mm-tall ring because of its relatively weak capability of confining the wave energy of designer surface plasmons.
